# Cell and Molecular Biology of Thyroid Disorders

**DOI:** 10.3390/ijms20122895

**Published:** 2019-06-13

**Authors:** Daniela Grimm

**Affiliations:** 1 Department of Biomedicine, Pharmacology, Aarhus University, Wilhelm Meyers Allé 4, 8000 Aarhus C, Denmark; 2 University Clinic for Plastic, Aesthetic and Hand Surgery, Otto von Guericke University, Leipziger Str. 44, 39120 Magdeburg, Germany; 3 Department of Microgravity and Translational Regenerative Medicine, Faculty of Medicine and Mechanical Engineering, Otto von Guericke University, Leipziger Str. 44, 39120 Magdeburg, Germany

The thyroid is a hormone gland involved in metabolism, regulatory functions, growth, and development of the human organism. The thyroid gland (glandula thyroidea) is butterfly-shaped and situated at the front of the neck, below the prominence of the Adam’s apple (thyroid cartilage). Its two side lobes lie around the trachea and are connected at the front by an isthmus—a narrow tissue strip (lobus pyramidalis) [1,2]. The main function of the thyroid gland is the storage of iodine, which is used for thyroid hormone production. The gland synthesizes and releases the thyroid hormones, triiodothyronine (T3) and thyroxine (T4), into the blood [3]. Hormone synthesis is regulated by thyroid-stimulating hormone (TSH), which is released by the anterior pituitary gland, which itself is regulated by thyrotropin-releasing hormone (TRH) produced by the hypothalamus. The parafollicular cells produce calcitonin, the peptide hormone, in response to high blood calcium levels.

The thyroid can be affected by various disorders. Hypothyroidism is the result of the insufficient synthesis and release of thyroid hormones. Iodine deficiency is the most common reason for hypothyroidism. Iodine deficiency can result in a goiter and cause uncomfortable symptoms like cardiac problems, pregnancy-related issues, and weight gain. It is the leading cause of preventable intellectual disability [4]. In iodine-sufficient regions, the most common cause of hypothyroidism is Hashimoto’s thyroiditis, which is an autoimmune disease [5]. Hyperthyroidism is the result of an excessive synthesis of thyroid hormones. The most common cause is the autoimmune disorder Graves’ disease [6]. The thyroid gland may also develop several types of nodules, benign tumors (adenomas), and cancer.

Thyroid cancer (TC) is the most common cancer of the endocrine system. TC is classified into several categories: (1) differentiated (DTC), covering papillary (PTC), follicular (FTC), and Hürthle cell cancer; (2) medullary (MTC); and (3) anaplastic thyroid cancer (ATC). In 2018, the American Cancer Society estimated that about 53,990 new cases of thyroid cancer were diagnosed (40,900 in women and 13,090 in men) and about 2060 deaths from TC (1100 women and 960 men) occurred in the United States. PTC comprises 80%–90% of all TC types [7]. Poorly differentiated thyroid cancer (PDTC) with increased dedifferentiation has a high risk of local recurrence, and is often characterized by a missing or insufficient uptake of radioiodine [8,9]. PDTC has a frequency of 10% in all thyroid cancer forms, is more aggressive than DTC, and has an unfavorable prognosis [8,9].

Recently, various studies have focused on improving TC diagnosis with the help of molecular biological techniques. Researchers searched for thyroid-specific biomarkers and focused on cancer-specific changes in gene expression and protein content using OMICS investigations [10,11,12,13,14]. In addition, new therapeutic agents, small molecules, and antibodies have been introduced and tested in vitro, in animals, and in clinical trials [8,9].

In this Special Issue, a total of 18 papers, consisting of 13 original articles and 5 reviews, have been published, as detailed in Table 1. This issue contains several manuscripts focusing on diagnosis [15], prognosis [16,17], and characterization [18,19,20] of TC, and anti-cancer drug (Multikinase inhibitor; MKI)-induced adverse effects in clinical trials [21]. Studies investigating the effects of microgravity on TC cells [22,23], reviews of mouse models for TC [24], and the aspects of the glycoprotein function in thyroid disorders [25] were published.

This Special Issue covers combined in vitro and animal studies [26,27] and single in vitro studies [28,29,30,31] investigating mechanisms or drug-induced changes in different thyroid cancer types. Proteomics technology was used to detect differences between the hypothyroid and euthyroid state in human subjects [32].

Rusinek et al. [15] reviewed the current advantages of preoperative molecular diagnostic tests and the histopathological examination of DTC. In addition, they discussed the DTC therapy, as well as new treatment modalities for radioiodine-refractory DTC. This review discusses data regarding ATC. Another focus was MTC. The authors reviewed clinical management based on histopathology and the ret proto-oncogene (RET) mutation genotype, follow-up, and the prognostic marker serum calcitonin, and finally the available targeted therapy in advanced MTC [15].

Milling et al. [21] reviewed tyrosine kinase-inhibitor (TKI) therapy in MTC, which is a rare malignancy with a poor prognosis. In non-operable cases or for patients with tumor progression and metastases, systemic treatment is necessary. MTC is often insensitive to conventional chemotherapy, but the use of TKI, such as pazopanib, cabozantinib, and vandetanib, has shown promising results with an increase in progression-free survival and prolonged lifetime [21], but TKI can cause various adverse events (AEs). One common AE of this treatment is hypertension, which can potentially influence the well-being of the treated patients. The authors discuss the available treatment strategies of drug-induced hypertension.

This Special Issue also covers original studies on patient and cell culture samples, providing novel mechanistic insights into TC pathogenesis and new aspects that may be important for the prognosis of TC or may impact diagnosis and clinical therapy. Despite low mortality, well-differentiated thyroid carcinomas (WDTC) frequently relapse [16]. B-Raf (rapidly accelerated fibrosarcoma) proto-oncogene (BRAF) and Telomerase Reverse Transcriptase (TERT) mutations are related to the prognosis in TC. The authors showed that PFKFB2 promoter methylation analysis has potential ability to better stratify WDTC patients according to recurrence risk, independently of BRAF and TERT mutations. They concluded that DNA methylation analysis of PFKFB2 promoter could be a potential tool for estimating the risk of recurrence in WDTC patients, that can be easily performed using a low-cost technique compatible with the clinical practice of bisulfite pyrosequencing.

Calabrese et al. [17] published in vitro data showing that miR-19a overexpression in FTC-133 cells induces a more de-differentiated and aggressive phenotype. miR-19a seems to be involved a poorer prognosis of thyroid cancer. The authors propose that this miRNA could represent a prognostic factor and a valid therapeutic target in highly malignant anaplastic tumors. Studies on human tissue samples have to be performed in the future to prove the association of miR-19a expression with the relevant clinicopathological factors and prognosis [17].

Rusinek et al. [18] reported, in a second research article, the coexistence of TERT promoter mutations and the BRAF V600E alteration and its impact on the morphology of papillary TC in Polish patient samples [18]. TERTp hotspot mutations were highly correlated with the presence of the BRAF V600E mutation [18]. Their coexistence was significantly associated with sex, advanced patient age and disease stage, lymph node metastases, increased tumor size, and tumor-capsule infiltration. Although correlations were identified, the possibility of TERTp mutations being key molecular modulators responsible for PTC aggressiveness requires further study [18].

Lee et al. [19] applied whole exome sequencing (WES) to identify a novel Hedgehog-interacting protein G516R mutation in tissue samples from three patients with locally advanced PTC. WES indicated intra-tumor heterogeneity in locally advanced thyroid cancers. This study first identified HHIP G516R (G1546A), which promotes tumor aggressiveness in TC cells. The authors used a new technique suitable for identifying new potential therapeutic targets for TC [19].

An ex vivo (patient tissue samples) and in vitro study investigating whether osteopontin-a (OPN; OPNa splice variant) overexpression is associated with matrix calcification in papillary TC was performed by Ferreira et al. [20]. OPN splice variants are often associated with tumor progression in PTC. PTC samples with psammoma bodies revealed an increased OPN expression level. OPNa overexpression promotes higher matrix calcification and collagen synthesis. In response to OPN knockdown, calcification was inhibited, in parallel with the downregulation of calcification markers. OPNa was the main contributor to matrix calcification in the tested samples, providing a better understanding of the biology and ethiopathogenesis of the calcification process in PTC [20].

Starenki et al. [31] showed an up-regulation of the mitochondrial heat shock protein 70 mortalin in FTC, PTC, and ATC tumor cells. Mortalin promotes survival and proliferation of the thyroid cancer cells. Application of triphenyl-phosphonium-carboxy-proxyl (Mito-CP) revealed that it can suppress ATC and PTC expressing RET/PTC or B-RafV600E.

Greco et al. [24] provide an overview of the imaging techniques used to date for both diagnosis and theranostic purposes in TC mouse models. These were developed to understand the fundamental mechanisms involved in tumorigenesis and to discover possible new targets. To date, many different approaches are available for imaging thyroid cancer in mouse models [24].

An in vivo and in vitro study focused on the impact of heme oxygenase-1 (HO-1) inhibitors on growth in mice tumor xenograft models and thyroid cancer cells [26]. HO-1 is overexpressed in thyroid cancer and is associated with tumor aggressiveness. The HO-1 inhibitors demonstrated therapeutic potential for inducing cell cycle arrest and promoting growth suppression of thyroid cancer cells [26].

Zhong et al. [27] investigated the anti-cancer effect of thiazolidinedione (TZD) and PPARγ agonist troglitazone and the statin lovastatin in human anaplastic thyroid cancer cells and in a mouse xenograft model [27]. The inhibitory effect of troglitazone/lovastatin is partly caused by cell cycle arrest (G0/G1 phase) and a decrease in hyperphosphorylated retinoblastoma protein signaling. These results support the hypothesis that the combination of troglitazone and lovastatin is a promising approach for treating ATC. The concept of statin-TZD therapy can be combined with other anti-cancer drugs [27].

In vitro studies have investigated the impact of simvastatin on anaplastic thyroid cancer cells [28]. The authors demonstrated that simvastatin inhibits the proliferation of RhoA/Rac1 protein by deactivation and overexpression of p21cip and p27kip, and reduced migration of the ATC cells [28].

Another in vitro study examined spheroids or thyrospheres, containing cancer stem-like cells, from B-CPAP and TPC-1 cell lines derived from PTC of the BRAF-like expression profile class, or stem-like cells from Nthy-ori 3-1 normal thyreocyte-derived cell line [29]. The authors recorded a significant decrease in glycolytic pathway metabolites and variations in Krebs cycle metabolites in three-dimensional (3D) aggregates versus parental cells. The authors demonstrated the metabolic profile of PTC cancer stem-like cells and suggested that metabolic changes are new biomarkers and targets for PTC therapy.

These findings are comparable to earlier data from studies investigating follicular thyroid cancer cells exposed to a Random Positioning Machine (RPM) [33]. Removing gravity forces resulted in changed concentrations of various glycolytic enzymes in FTC-133 follicular thyroid cells. Western blot analysis and flow cytometry revealed, for example, a reduction in alpha-enolase in FTC-133 follicular thyroid cancer cells grown for three days under microgravity conditions compared to static 1 *g* -samples [33].

A comparative proteome analysis of FTC-133 thyroid cancer cells, growing as a monolayer under normal gravity or within 3D spheroids under simulated microgravity realized by an RPM, showed an up-regulation of 69 proteins detected in spheroids [22]. Using semantic and in silico analyses, the authors showed that a high percentage of the 69 selected proteins had modifiable N6 lysine residues. This study shows a novel method to facilitate planning work on possible posttranslational modifications (PTMs) of the proteins of cells, actually changing their type of growth, and offers explanations for earlier results regarding the protein-lysine 6-oxidase (*LOX*) gene. A tremendous down-regulation of the *LOX* gene was observed in FTC-133 cells during the Shenzhou-8/SimBox space mission [34,35]. The protein-lysine 6-oxidase catalyzes deamination of lysine residues [36].

Krüger et al. [23] reviewed the current knowledge about TC and microgravity research. Microgravity influences processes such as apoptosis, the cytoskeleton, adhesion, the extracellular matrix, and influences cell growth [23,37]. FTC cells exposed to microgravity conditions shifted toward a less-malignant phenotype. Results from space medicine are important for rethinking conventional cancer research and may help pinpoint the cellular changes causing cancer. This knowledge may help develop novel therapies that will enhance the quality of life for patients or potentially help develop new countermeasures [23].

A further in vitro study focused on the impact of the mitogen-activated protein kinase (MEK) inhibitor selumetinib in different thyroid carcinoma cell lines [30]. Selumetinib significantly reduced cell viability. The drug restored the sodium iodide symporter (NIS) by inhibition of its related targeting miRNAs. Future studies to clarify the mechanism activated by hsa-miR-146b-5p, hsa-miR-146b-3p, and hsa-let7f-5p to stabilize NIS should be performed. Restoration of the NIS might be a new option to treat advanced radioiodine refractory differentiated TC [30].

This Special Issue also covers benign thyroid disorders. One study investigated the differences in the plasma proteome of patients with hypothyroidism before and after thyroid hormone substitution therapy [32]. Proteomics technology was applied to compare the plasma proteome between the hypothyroid and the euthyroid states in patients [32]. Changes in the expression of several acute-phase response proteins were detected. A pathway analysis revealed interleukin-6 and tumor necrosis factor- α (TNF-α) as central factors and as dysregulated in hypothyroidism.

The aspects of glycoprotein functioning in thyrocyte physiology and thyroid disorders were reviewed for this issue [25]. Changes in glycan structures result in the progression of thyroid cancer and autoimmunity. Tumorigenesis is accompanied by changes in sialylation and fucosylation, β1,6-branching of glycans, the content and structure of poly-LacNAc chains, as well as O-GlcNAcylation. In thyroid autoimmunity, the main processes affected are sialylation and fucosylation. Thyroid glycobiology helps us to understand the role of glucose in disorders [25].

Overall, the 18 important contributions published in this Special Issue demonstrate novel findings in the field of thyroid research. I thank all the authors who contributed to this Special Issue, and I remain hopeful that the application of new molecular biological technologies will help with benign and malignant thyroid disorders and that the increasing knowledge of diagnosis, prevention, and new treatment strategies for TC, as well as the search for new proteins that may serve as new drug targets, will help reduce the incidence and mortality of advanced metastatic TC.

## Figures and Tables

**Table 1:** Contributions to the Special Issue “Cell and Molecular Biology of Thyroid Disorders”.

Author	Title	Topics	Type	Reference
Rusinek et al.	Current advances in thyroid cancer management. are we ready for the epidemic rise of diagnoses?	Diagnosis of TC	Review	[15]
Chen et al.	Simvastatin inhibits cell proliferation and migration in human anaplastic thyroid cancer	ATC: Drug testing in vitro	Research article	[28]
Greco et al.	Preclinical imaging for the study of mouse models of thyroid cancer	Imaging techniques used for diagnosis in TC mouse models	Review	[24]
Alfadda et al.	Differences in the plasma proteome of patients with hypothyroidism before and after thyroid hormone replacement: a proteomic analysis	Plasma proteome-hypothyroid vs. euthyroid state	Research Article	[32]
Zhong et al.	A synergistic anti-cancer effect of troglitazone and lovastatin in a human anaplastic thyroid cancer cell line and in a mouse xenograft model	ATC: Drug testing in vitro	Research Article	[27]
Yang et al.	Heme oxygenase-1 inhibitors induce cell cycle arrest and suppress tumor growth in thyroid cancer cells	FTC, undifferentiated TC: Drug testing in vitro	Research Article	[26]
Wächter et al.	Selumetinib activity in thyroid cancer cells: modulation of sodium iodide symporter and associated miRNAs	ATC, PTC and undifferentiated TC: Drug testing in vitro	Research Article	[30]
Bauer et al.	Semantic analysis of posttranslational modification of proteins accumulated in thyroid cancer cells exposed to simulated microgravity	FTC: Proteomics	Research Article	[22]
Rusinek et al.	Coexistence of TERT Promoter mutations and the BRAF V600E alteration and its impact on histopathological features of papillary thyroid carcinoma in a selected series of Polish patients	PTC: diagnosis	Research Article	[18]
Ząbczyńska et al.	Glycosylation in the thyroid gland: vital aspects of glycoprotein function in thyrocyte physiology and thyroid disorders	Changes in protein glycosylation profiles lead to thyroid disorders	Review	[25]
Vilsbøll Milling et al.	Pazopanib, cabozantinib, and vandetanib in the treatment of progressive medullary thyroid cancer with a special focus on the adverse effects on hypertension	ATC: Therapy	Review	[21]
Lee et al.	Whole exome sequencing identifies a novel hedgehog-interacting protein g516r mutation in locally advanced papillary thyroid cancer	Whole Exome Sequencing for advanced PTC	Research Article	[19]
Ferreira et al.	OPNa overexpression is associated with matrix calcification in thyroid cancer cell lines	PTC: diagnosis	Research Article	[20]
Caria P. et al.	Metabolomic alterations in thyrospheres and adherent parental cells in papillary thyroid carcinoma cell lines: a pilot study	PTC: in vitro study	Research Article	[29]
Calabrese et al.	miR-19a overexpression in FTC-133 cell line induces a more de-differentiated and aggressive phenotype	FTC: in vitro study	Research Article	[17]
Barros-Filho et al.	PFKFB2 promoter hypomethylation as recurrence predictive marker in well-differentiated thyroid carcinomas	DTC: Recurrence Predictive Marker	Research Article	[16]
Starenki et al.	Mortalin (GRP75/HSPA9) promotes survival and proliferation of thyroid carcinoma cells	PTC, FTC, ATC express high mortalin	Research Article	[31]
Krüger et al.	Fighting thyroid cancer with microgravity research	Microgravity alters apoptosis, adhesion, proliferation, the cytoskeleton and the extracellular matrix. FTC cells grown in space were shifted towards a less-malignant phenotype.	Review	[23]

## Abbreviations

AE	Adverse events
ATC	Anaplastic thyroid cancer
*BRAF*	B-Raf (rapidly accelerated fibrosarcoma) proto-oncogene
cfDNA	Cell-free deoxyribonucleic acid
DTC	Differentiated thyroid cancer
EGFR	Epidermal growth factor receptor
FNA	Fine needle aspiration
FNAC	Fine needle aspiration cytology
FTC	Follicular thyroid cancer
HER2	Human epidermal growth factor receptor 2
HO1	Hemoxygenase 1
MKI	Multi-kinase inhibitors
MTC	Medullary thyroid cancer
miRNA	Micro-ribonucleic acid
OPN	Osteopontin
PTC	Papillary thyroid cancer
PTK2/FAK1	Focal adhesion kinase 1
PTM	Posttranslational modification
PXN	Paxillin
RAS	Rat sarcoma proto-oncogene
RET	Ret proto-oncogene
RPM	Random positioning machine
TC	Thyroid cancer
TERT	Telomerase reverse transcriptase
TKI	Tyrosine-kinase inhibitors
*TNFSF4*	Tumour necrosis factor superfamily member 4
TNM	TNM Classification of malignant tumours
TSH	Thyroid-stimulating hormone
TTF1	Thyroid transcription factor 1
VCL	Vinculin
